# Comparative RNAseq Analysis of the Insect-Pathogenic Fungus *Metarhizium anisopliae* Reveals Specific Transcriptome Signatures of Filamentous and Yeast-Like Development

**DOI:** 10.1534/g3.120.401040

**Published:** 2020-04-28

**Authors:** Natasha Sant’Anna Iwanicki, Italo Delalibera Júnior, Jørgen Eilenberg, Henrik H. De Fine Licht

**Affiliations:** * Department of Entomology and Acarology, ESALQ- University of São Paulo, Av Padua Dias, 11–P.O. Box 9–13418-900, Piracicaba, SP, Brazil and; ^†^ Department of Plant and Environmental Sciences, University of Copenhagen, Thorvaldsensvej 40, 1871 Frederiksberg C, Denmark

**Keywords:** Fungal morphogenesis, Entomopathogenic fungi, Hypocreales, Differentially expressed genes (DEGs), Blastospores

## Abstract

The fungus *Metarhizium anisopliae* is a facultative insect pathogen used as biological control agent of several agricultural pests worldwide. It is a dimorphic fungus that is able to display two growth morphologies, a filamentous phase with formation of hyphae and a yeast-like phase with formation of single-celled blastospores. Blastospores play an important role for *M. anisopliae* pathogenicity during disease development. They are formed solely in the hemolymph of infected insects as a fungal strategy to quickly multiply and colonize the insect’s body. Here, we use comparative genome-wide transcriptome analyses to determine changes in gene expression between the filamentous and blastospore growth phases *in vitro* to characterize physiological changes and metabolic signatures associated with *M. anisopliae* dimorphism. Our results show a clear molecular distinction between the blastospore and mycelial phases. In total 6.4% (n = 696) out of 10,981 predicted genes in *M. anisopliae* were differentially expressed between the two phases with a fold-change > 4. The main physiological processes associated with up-regulated gene content in the single-celled yeast-like blastospores during liquid fermentation were oxidative stress, amino acid metabolism (catabolism and anabolism), respiration processes, transmembrane transport and production of secondary metabolites. In contrast, the up-regulated gene content in hyphae were associated with increased growth, metabolism and cell wall re-organization, which underlines the specific functions and altered growth morphology of *M. anisopliae* blastospores and hyphae, respectively. Our study revealed significant transcriptomic differences between the metabolism of blastospores and hyphae. These findings illustrate important aspects of fungal morphogenesis in *M. anisopliae* and highlight the main metabolic activities of each propagule under *in vitro* growth conditions.

Fungi show a high degree of phenotypic plasticity with fungal cells exhibiting a diverse array of shapes and sizes. Part of this plasticity in fungal phenotypes is a consequence of the indeterminate growth of fungi, but also manifests itself as polyphenisms in the form of discrete and distinct types of fungal structures such as hyphae, conidia, dividing single-cells etc. The various types of fungal structures are important at different stages of fungal growth and reproduction, and especially transitions between yeast-hyphal dimorphisms are significant for the virulence of dimorphic pathogenic fungi (Moore 1998). For the human fungal pathogens *Histoplasma capsulatum* and *Paracoccidioides brasiliensis*, the yeast phase is involved in the infection processes (Felipe *et al.* 2003, Hwang *et al.* 2003) whereas both the yeast and mycelium phases are involved in pathogenic growth in the human pathogen *Candida albicans* and the plant pathogen *Ophiostoma novo-ulmi* (Gow *et al.* 2002, Comeau *et al.* 2014). However, our understanding of the underlying genetic mechanisms of how fungal pathogens switch between phenotypes and growth forms is rather limited and primarily restricted to a few mostly human pathogenic fungi where fungal dimorphism is thermally regulated.

Fungi from the genus *Metarhizium* are highly diverse and many show a remarkable degree of phenotypic plasticity. Depending on external stimuli and exact species involved, *Metarhizium* can grow inside insects as entomopathogens (Shahid *et al.* 2012, Boomsma *et al.* 2014), inside plants as primarily root endophytes (Elena *et al.* 2011, Sasan and Bidochka 2012), or as hyphae in the soil connecting insect carcasses and plants (Behie *et al.* 2012). The species *M. anisopliae* and *M. brunneum* are widely used as biological control agents against pest insects and mites. The fungi are cultivated on solid substrates, such as rice, for 10-14 days until infectious, uniform, hydrophobic conidia can be harvested and formulated into a dry powder-based product. This is not very cost-effective and for several fungi employed as biological control agents, submerged culture fermentation has been considered as a more optimal method for the production of infective propagules (Jackson *et al.* 1997 and Mascarin *et al.* 2015a).

In addition to hyphae and conidia, *Metarhizium* morphogenesis also includes microsclerotia and blastospores. The former is an overwintering structure made of often melanized compact hyphal aggregates that can be induced in carbon-rich submerged liquid cultures (Jaronski and Mascarin (2016) and Mascarin *et al.* 2014). Microsclerotia are considered desiccation tolerant and able to produce infective conidia at specific environmental conditions when applied in the field (Goble *et al.* 2017). Blastospores are thin-walled, pleomorphic, hydrophilic single fungal cells that can be induced after only 2-3 days of liquid fermentation. Following germination, both conidia and blastospores are able to penetrate insect cuticle using mechanical and enzymatic force or via natural openings of the insects (Alkhaibari *et al.* 2016, Bernardo *et al.* 2018). Once inside the insect, *Metarhizium* proliferates in the hemocoel as single-celled yeast-like structures that are also termed blastospores. Compared to aerial conidia, blastospores have been found to be more virulent against susceptible hosts (Mascarin *et al.* 2015, Alkhaibari *et al.* 2016, Bernardo *et al.* 2018), although less desiccation tolerant (Jaronski and Mascarin (2016). This makes blastopores an attractive but tricky alternative to conidia for biological control although it is unclear exactly why blastospores are more virulent. Blastospores generally germinate within 2-8 hr compared to 12-24 hr for conidia (Alkhaibari *et al.* 2016, Bernardo *et al.* 2018), which is an attractive trait for applied purposes and could explain the increased virulence compared to conidia.

In the present study, we aimed to investigate physiological changes and metabolic signatures of the *M. anisopliae s. str*. growth phases, blastospores and hyphae. Specifically, we hypothesized whether changes in gene expression can be related to i) phenotypic differentiation and growing processes in hyphae and blastospores, ii) potential differences in fungal cell-wall metabolism, iii) responses in cellular respiration and oxidative stress to liquid and solid media, iv) genes involved in arthropod pathogenicity that are differentially expressed between hyphae and blastospores, and v) specific classes of biosynthesis genes involved in secondary metabolism produced by each fungal structure.

## Material and Methods

### Fungal material and laboratory culturing

The present study investigated the fungus *Metarhizium anisopliae* sensu stricto (ss) using a strain (ESALQ4676) isolated from soil of a rainforest biome in native vegetation in Alagoas state, 9 51’7,30”S, 36 20’0,40”W in Brazil. The fungus was isolated using an insect-baiting method using *Galleria melonella* (Lepidoptera: Pyralidae) as insect bait. A conidial monospore culture was obtained by growing the fungus in potato dextrose agar (PDA, Difco, Sparks, MD, USA) for three weeks in a growth chamber at 26° and 12:12 h photoperiod. A fungal stock culture was established by preserving sporulating agar chunks immersed in a sterile 10% glycerol solution at -80°. The isolate is deposited in the Entomopathogenic Fungal Collection at ESALQ-University of São Paulo (Piracicaba, Brazil) with the accession number ESALQ4676.

**Figure 1:**
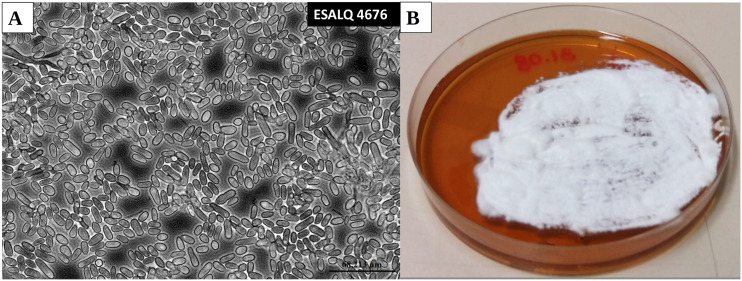
Phase-contrast microscopic image of *Metarhizium anisopliae* (ESALQ4676) blastospores produced in liquid culture (4 days of culture) (magnification: 400x) (a) and mycelium of *M. anisopliae* grown in modified Adamek medium (5 days of growing) (b).

Fungi were grown on a modified Adamek medium (Iwanicki *et al.* 2018) with the following nutritional composition per liter: 80 g yeast extract, 40 g cornsteep liquor (Sigma, St. Louis, USA), minerals, trace metals and vitamins adapted from Jackson’s medium (Jackson *et al.* 1997) at the following concentrations per liter: KH_2_PO_4_, 2.5 g; CaCl_2_0.2H_2_O, 1.0 g; MgSO_4_0.7H_2_O, 0.83 g; FeSO_4_0.7H_2_O, 0.3 g; CoCl_2_0.6H_2_O, 29.6 mg; MnSO_4_.H_2_O, 12.8 mg; ZnSO_4_0.7H_2_O, 11.2 mg; 0.2 mg each of thiamin, riboflavin, pantothenate, niacin, pyridoxamine, thioctic acid; and 0.02 mg each of folic acid, biotin, and vitamin B12. The medium was amended with 140g L^-1^ of glucose solutions that were autoclaved separately. Sterile solutions of vitamins and metals were added to the autoclaved medium before pH was adjusted to 6.8. Growth and formation of mycelial hyphae during the filamentous phase of the fungus (Figure 1a) and hyphal bodies (blastospores) in the single-celled yeast-like state (Figure 1b) where induced by growing *M. anisopliae* ESALQ4676 on solid and in liquid modified Adamek medium, respectively. Thus identical nutritional compositions were used to grow mycelial hyphae and blastospores, with the only difference that 15 g L^-1^ of agar were added to solidify the medium used for inducing hyphal growth.

### Induction of hyphal and blastospore formation in vitro

Conidia of *M. anisopliae* ESALQ4676 were obtained by growing agar chunks from the monosporic stock culture in Petri dishes containing potato dextrose agar in a concentration of 39g per liter of distilled water (PDA, Difco, Sparks, MD, USA) for ten days at 26° and 12:12 h photoperiod. Conidia were harvested by washing Petri dishes containing actively sporulating fungal cultures with 10 mL of a sterile aqueous solution with 0.02% polyoxyethylene sorbitan monooleate (Tween 80, Sigma). A conidial suspension of 5×10^6^ conidia mL^-1^ was used to inoculate 50 mL liquid modified Adamek medium in 250-mL baffled Erlenmeyer flasks giving a final concentration in the culture broth of 5×10^5^ conidia mL^-1^ (*i.e.*, 10% v/v inoculum). Liquid cultures were incubated at 28° in a rotatory incubator shaker and 350 rpm for 72 hr. Four Erlenmeyer flasks were inoculated and each considered a biological replicate. A volume of 120uL of the 5×10^6^ conidia mL^-1^ suspension was spread with sterile Drigalski handles in Petri dishes (9 cm diameter) containing solid modified Adamek medium. Four fungal cultures on solid media in separate petri dishes were incubated at 28° for 5 days with a 12:12 h photoperiod, and each considered as one biological replicate.

### RNA extraction and sequencing

Blastospores were harvested by filtering three-day old fungal cultures grown in liquid modified Adamek medium. The time point were chosen because pilot laboratory experiments established that the blastospore population was at the end of the exponential growth phase, but it was possible to obtain large amount of cells for extraction. Total culture broth of each replicate were filtered in a vacuum pump coupled to a Buchner funnel lined with disk filter paper with 7 cm diameter and 11µ pore sizes (Whatman, n°1) to remove hyphae. To verify that the filtrate contained only blastospores, each replicate was examined on microscope slides at 400x magnification using optic microscopy. To separate blastospores from the culture medium, 30mL of filtrate were added to 50mL Falcon tubes and centrifuged at 2500 rpm, 4°. for 5 min. The supernatant was discarded and the blastospore pellet was quickly transferred with a pre-cooled scoop to a pre-cooled porcelain mortar, before immediately adding liquid nitrogen and macerating the pellet with a pre-cooled pestle. The resulting powder were not allowed to thaw and transferred to an Eppendorf tube containing 1mL of TRIzol and kept on ice. A pilot study was carried out to determine the moment of expressive mycelial growth without the presence of conidia in agar medium, to determine optimal time point but for RNA extraction. Then, mycelial hyphae were harvested from five-day old fungal cultures grown on modified Adamek agar media (Iwanicki *et al.* 2018). Hyphae were removed from the medium with a sterilized and pre-cooled spatula and placed immediately in an Eppendorf tube containing 1mL of TRIzol and kept on ice. Care was taken to avoid collecting the modified Adamek medium when scraping off the hyphae.

Total RNA was extracted from fungal samples immersed in TRIzol reagent (Invitrogen, USA) following the manufacturer’s instructions. Eppendorf tubes containing 1mL of TRIzol with either blastospores or hyphae were incubated for 5 min at room temperature, before homogenizing the samples by pipetting up and down. This was followed by centrifugation for 5 min at 12000x G at 4°. The supernatant was transferred to a new clean Eppendorf tube and the samples were homogenized for 5 min in a tissue homogenizer to break the fungal cell walls. 200µL of chloroform were added to samples following agitation for 15 sec and incubated at room temperature for 5 min. Then, another centrifugation was performed (12,000 × spin, 15 min at 4°) to separate the mixture and total RNA was precipitated from the upper aqueous phase with half a volume isopropanol (0.5 ml isopropoanol per 1 mL of TRIzol) and centrifugation. The pellet was washed with 1 mL of 75% ethanol and placed to dry for 30 min at room temperature, followed by resuspension of total RNA in 20 uL of pre-cooled DEPC-treated water. Total RNA was quantified fluorometrically using a Qubit (Invitrogen) and the purity and quality evaluated in a NanoDrop ND-1000 spectrophotometer (Wilmington, USA). The RNA integrity was estimated with 1% agarose-formaldehyde gel capillary electrophoresis using a Bioanalyzer (Agilent), and only samples with a RNA integrity measure (RIN) higher than 8 were used.

Messenger RNA libraries were prepared with Illumina TruSeq Stranded mRNA Library Prep kit (Illumina Inc., San DieGo, CA) and quantified with qPCR using the Illumina KAPA Library Quantification kit. Samples were sequenced with Illumina HiSeq 2500 technology, which yielded at least 20 million 100-bp paired-end reads per library. Library preparation and sequencing were performed by “Laboratório Multiusuários Centralizado de Genômica Funcional Aplicada à Agropecuária e Agroenergia” in Piracicaba-SP, Brazil.

### Mapping of RNA-Seq reads and quantitative differential expression analysis

**Figure 2:**
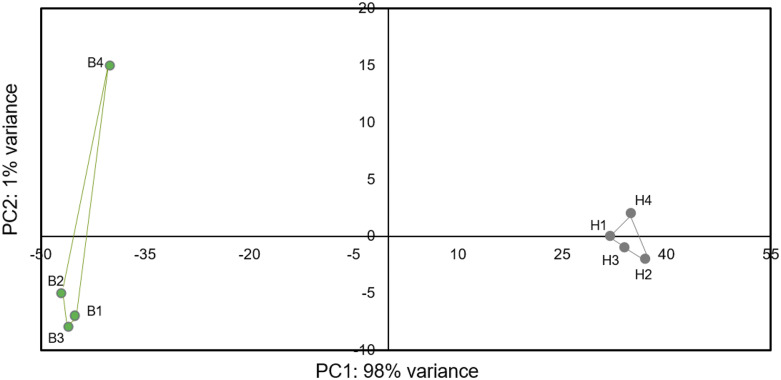
Principal component analysis of regularized-logarithmic (rlog) transformed gene counts of blastospores (B1-4) and hyphal samples (^1^H-4). Blastospores and hyphae samples are represented by green and gray dots, respectively.

The quality of the raw reads before and after quality and adaptor trimming was assessed using the fastQC (Simon) program. Illumina adapters and low-quality sequences were removed using Trimmomatic V0.32 (Bolger *et al.* 2014) with the following options: HEADCROP:7 TRAILING:20 MINLEN:36. Quality trimmed reads were aligned to the reference genome (*M. anisopliae* sensu stricto ARSEF549 from NCBI) using HISAT2 (version 2.0.1) (Pertea *et al.* 2016). First, we used the python scripts included in the HISAT2 package: extract_splice.py and extract_exons.py, to extract the splice-site and exon information’s from the annotation file, respectively. Then, we built the indexes for the reference genome with the program hisat2-build with the options:–ss and –exon, to provide outputs from splice sites and exons, respectively. Finally, we aligned RNA-seq reads to the reference genome with the program hisat2 with the options: –dta and –p 8. Gene quantification were performed with StringTie v1.3.3 (Pertea *et al.* 2016) using gene annotations from *M. anisopliae* ARSEF549 strain reference genome information. The stringtie program were used with the following options: -b, -B and –G. The gene count matrix were obtained with the python script: prepDE.py, provided by John Hopkins University, center for computational biology, CCB (http://ccb.jhu.edu/software/stringtie/index.shtml?t=manual#deseq). The gene count matrix was used as input file to the differential expression analysis that was conducted using DESeq2 Love *et al*. (2014) from the statistical software R (version 3.6) (R Core Team 2017) We chose a conservative approach to designate differential expression to avoid false positives, so only genes with a false discovery rate (FDR) adjusted p-values <0.001 and log_2_ fold change (FC) > 4, for up-regulated genes and log_2_FC < -4, for down-regulated genes were considered differentially expressed. Genes were considered exclusively expressed in either blastospores or hyphae when all the biological samples from one fungal structure showed expression values in the same direction (up or down), while all biological replicate samples for the other fungal structure had no reads mapped to that same gene. Diagnostic plots (MA-plot and Volcano-plot) are provided in supplementary material (Additional file 10). Individual gene expression was not re-validated by qPCR because previous studies have shown extremely close correlation between qPCR and RNAseq data (Asmann *et al.* 2009, Griffith *et al.* 2010, Wu *et al.* 2014, Shi and He 2014), our biological samples are robustly replicated and being highly similar within treatments and clearly distinct between treatments (Figure 2), and there is little evidence that qPCR analyses of a few specific genes of the same samples will add any new utility to our data or change the major conclusions drawn from the much larger groups of genes analyzed in the RNAseq dataset. Heatmaps of differentially expressed genes were made with the web application “shinyheatmap” (Khomtchouk *et al.* 2017) with the following parameters: apply clustering: column, Distance metric: Euclidian; Linkage algorithm: complete.

### Gene-set enrichment analysis

Gene set enrichment analysis (GSEA) is a software that determines whether *a priori* defined set of genes is statistically significant between two biological states (Subramanian *et al.* 2005). GSEA rank genesets by enrichment magnitude and indicate classes of genes that are over-represented in geneset. As recommended for RNA-seq datasets, GSEA was used in the GSEAPreranked mode with a user provided list of all genes pre-ranked according to a defined metric that could be the log_2_ fold change, adjusted *p-value* or inverse *p-value* and a list of gene sets. Then, GSEAPreranked calculates an enrichment score by matching genes from gene sets to those in the user ranked list. Next, the gene set’s enrichment score shows how often members of that gene set occur at the top or bottom of the ranked data set. In this study, we used GSEAPreranked mode with gene sets categorized by gene ontology (GO), protein family domain (PFAM), and Kyoto Encyclopedia of Genes and Genome (KEGG) pathway annotation. The metric used in GSEA input file was the multiplied the sign of fold change by its inverse *p-value*. We used the *p-value* provided as an output of DESeq2. When the *p-value* output from DESeq2 was “0”, the “0” value was replaced by artificially high or low values “+1E+308” or “-1E+308” for up and down-regulated genes, respectively, according to the sign of fold change. The parameter adopted for running the GSEAPreranked for KEGG, GO and PFAM terms were: minlength 10 and maxlength 500, enrichment statistic: “classic” and FDR-correction for multiple testing < 0.25 for enriched gene sets. The unusual high FDR threshold of < 0.25 is recommended by GSEA because it indicates that the result is likely to be valid 3 out of 4 times, which arguably is reasonable for exploratory discovery analysis before future validation (Subramanian *et al.* 2005). The web server REVIGO (Supek *et al.* 2011) was used to analyze GO terms in the categories: *biological process*, *cellular component* and *molecular function*.

### Secondary metabolites

We identified genes from different families of secondary metabolism pathways classified by Donzelli and Krasnoff (2016), which are based on orthology, phylogenetic analysis and conservation of gene organization around them. The following families were investigated: Nonribosomal peptide synthetase pathway (NRPS), Polyketides synthases (PKS), Hybrid PKS-NRPS (HPN) and Terpenoids biosynthesis (TER). Genes differentially expressed between blastospores and hyphae were filtered by false discovery rate (FDR) adjusted *p-values* were <0.001 and log_2_ fold change (FC) > 4 or -4 as described above. Functional annotation of differentially expressed genes according to Donzelli and Krasnoff (2016) are provided Additional file 9.

### Data availability

The transciptome metadata generated during the current study are available in the European Nucleotide Archive repository, [https://www.ebi.ac.uk/ena, under the accession number: PRJEB30948].

Additional file 1. Complete dataset of the gene differential expression analyzes and its statistics for all genes mapped against the *Metarhizium anisopliae* genome ARSEF 549.
Additional file 2. Dataset with significantly differentially expressed genes. Spreadsheet 1: Genes up- regulated in blastospores (Log2FC >4, padjust < 0.001) Spreadsheet 2: Genes down-regulated in blastospores (Log2FC < -4, padjust < 0.001)
Additional file 3. Dataset with genes exclusively expressed in hyphae (Spreadsheet 1) or blastospores (Spreadsheet 2)
Additional file 4. Enriched Gene ontology (GO), Protein families (PFAM) and KEGG (ko pathway) terms in hyphae (Spreadsheet 1) and blastospores (Spreadsheet 2) resulted from Gene set enrichment analysis.
Additional file 5. Dataset with enriched PFAM and KEGG that occur exclusively in hyphae (Spreadsheet 1) or blastospores (Spreadsheet 2) within the up or down-regulated genes
Additional file 6. Genes grouped by Glycoside hydrolase (GH) family and protein family
Additional file 7. Dataset with top 50 genes up and down-regulated
Additional file 8. Dataset with specific protein families involved in virulence factors
Additional file 9. Dataset with families of biosynthetic genes involved in secondary metabolism of *Metarhizium*
Additional file 10. Diagnostic plots of RNA-Seq data: MA-Plot and Volcano-plot
Additional file 11. Genes not expressed in either phase. Supplemental material available at figshare: https://doi.org/10.25387/g3.12159834.

## Results

### RNAseq data statistics and reproducibility

To compare genome-wide expression profiles of blastospores and hyphae of *M. anisopliae* (ESALQ4676), a total of 115 million paired-end 100-bp quality-checked reads from four replicate blastospores and four replicate hyphal samples (between 13-16 million paired-end reads per sample), were obtained. The percentage of quality filtered reads that mapped to the *M. anisopliae* reference genome (ARSEF549) were 94% for blastospores and 84% for hyphae (Table 1). Clustering analysis showed that 98% of sample variation was represented by differences between treatments and consequently a high similarity of biological samples within treatment (blastospores *vs.* hyphae) (Figure 2).

**Table 1:** Summary of *M. anisopliae* RNA-Seq read filtering and mapping. Values represents the values for each of the four biological replicates for blastospores (BL) and hyphae (H), respectively

Sample	Clean paired reads	Mapped reads (%)	Unmapped reads (%)	Unique match (%)
**BL1**	15,949,141	15,049,609 (94.3%)	899,531 (5.7%)	9011677 (56%)
**BL2**	14,305,193	13,555,600 (94.7%)	749,592 (5.3%)	8079589 (56.4%)
**BL3**	13,170,251	12,466,959 (94.6%)	703,291 (5.4%)	8022903 (60.9%)
**BL4**	16,858,276	15,990,074 (94.8%)	868,201 (5.2%)	8704384 (51.63%)
**H1**	13,555,682	11,481,662 (84.7%)	2074,019 (15.3%)	7258528 (53.54%)
**H2**	13,470,151	11,320,314 (84%)	2149,836 (16%)	7007145 (52.09%)
**H3**	14,050,046	11,872,288 (84.5%)	2177,757 (15.5%)	7255368 (51.63%)
**H4**	13,988,895	11,673,732 (83.4%)	2315,162 (17.6%)	7410046 (52.9%)

**Figure 3:**
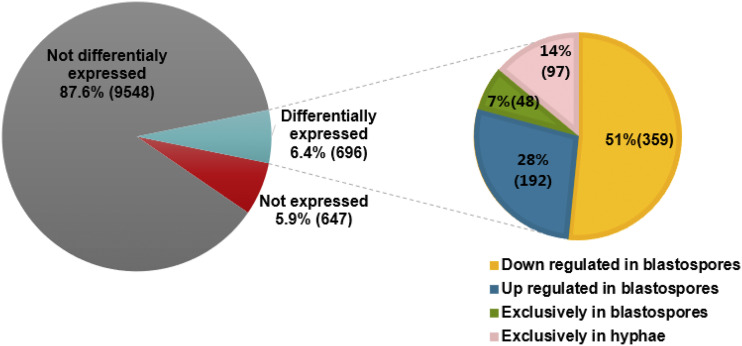
Number of genes differentially expressed in blastospores *vs.* hyphae. The percentage of genes that are not expressed, not differentially expressed and differentially expressed are shown in the left circle. The right circle shows the percentage of down and up-regulated genes in blastospores and genes exclusively expressed in blastospores or hyphae out of the differentially expressed genes.

**Figure 4:**
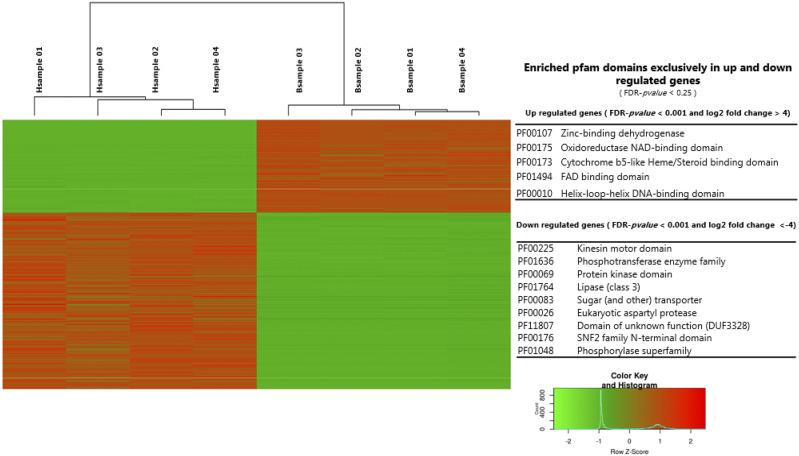
Heat map of the 696 genes differentially expressed (FDR-*pvalue* < 0.001 and log2 fold change > 4 or -4), with 240 up and 456 down-regulated genes in blastospores, respectively. The enriched exclusively pfam domains assigned to up and down-regulated genes are presented in the right panel (Additional file 3).

From 10,891 genes annotated in the *M. anisopliae* genome (ARSEF 549) (Additional file 1), 696 genes were differentially expressed between blastospores and hyphae (FDR adjusted *P* < 0.001, Log_2_FC > 4 or < -4). Of these, 240 genes were up-regulated in blastospores and 456 were up-regulated in hyphae (Figure 3, 4 and Additional file 2). Moreover, we found 48 genes exclusively expressed in blastospores, and 97 genes exclusively expressed in hyphae (Additional file 3). A set of 647 genes in the reference genome were not expressed in any of the two fungal growth phases analyzed here.

### Gene set enrichment analyses

**Figure 5:**
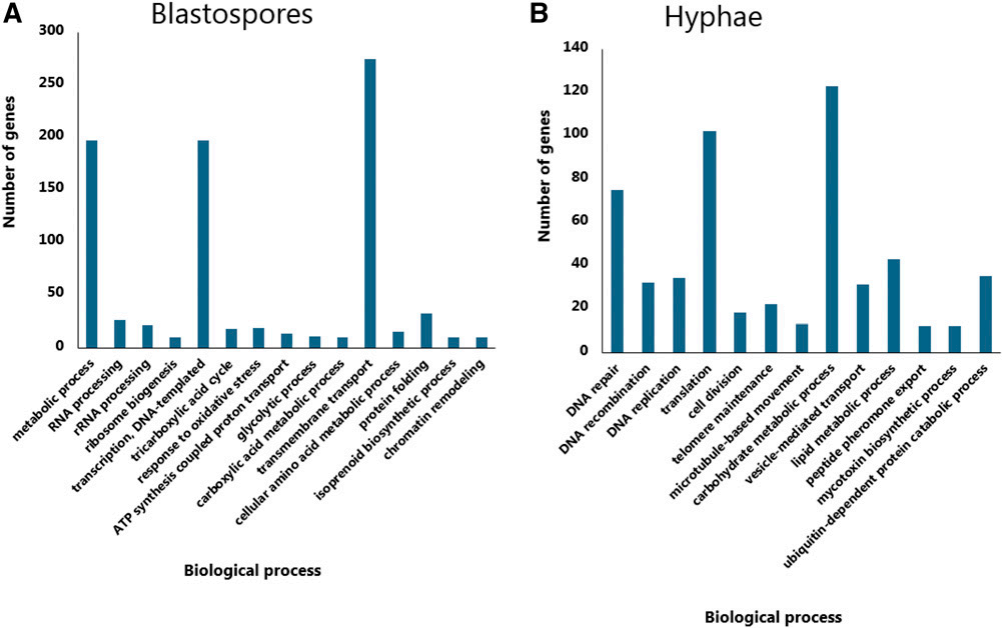
Enriched gene ontology (GO) terms for the *biological process* category based on GSEA analysis (See text for details) in blastospores (5a) and hyphae (5b) (FDR- *p-value* < 0.25) (Additional file 4).

Gene Ontology (GO) terms could be assigned to 66% (465/696) of the differentially expressed genes. To functionally characterize the set of genes significantly up-regulated in blastospores and hyphae we carried out GO-term gene set enrichment analyses using GSEA (Subramanian *et al.* 2005). In blastospores, 69 GO terms were significantly enriched (FDR adjusted *P* < 0.25), of these, 15 were assigned to biological processes, eight to cellular components and 46 to molecular function (Additional file 4). The largest set of enriched genes among the biological processes were assigned to: *metabolic process* (GO:0008152), *Transcription*, *DNA-templated* (GO:0006351), and *transmembrane transport* (GO:0055085) (Figure 5a). Thirty-three GO terms were found to be significantly enriched in hyphae compared to blastospores (FDR adjusted *P* < 0.25), with 13 assigned to biological processes (Figure 5b), five to cellular components and 15 to molecular functions (Additional file 4).

Pfam terms could be assigned to 61% (427/696) of differentially expressed genes. Of the total differentially expressed genes, we found an overlap of 362 genes that could be assigned to GO and Pfam terms. Those genes are listed in Additional file 4 in the spreadsheet named: “Overlap_Genes_assignGO_and_PFAM”. Using the same GSEA-methodology as described above, 28 pfam terms were significantly enriched in blastospores (Additional file 4). Five of these, (Zinc-binding dehydrogenase (PF00107), Oxidoreductase NAD-binding domain (PF00175), Cytochrome b5-like Heme/Steroid binding domain (PF00173), FAD binding domain (PF01494), and Helix-loop-helix DNA-binding domain (PF00010) are exclusively found among up-regulated genes in blastospores (Figure 4 and Additional file 5). A total of 18 pfam terms were significantly enriched among up-regulated genes in hyphae (Additional file 4), of which nine were exclusively present among the up-regulated genes in hyphae (PF00225:Kinesin motor domain, PF01636:Phosphotransferase enzyme family, PF00069:Protein kinase domain, PF01764:Lipase (class 3), PF00083:Sugar (and other) transporter, PF00026:Eukaryotic aspartyl protease, PF11807:Domain of unknown function (DUF3328), PF00176:SNF2 family N-terminal domain, PF01048:Phosphorylase superfamily)(Figure 4 and Additional file 5). To explore patterns of metabolic pathway regulation between blastospores and hyphae we attempted to assign KEGG terms, which resulted in KEGG annotation of 15% (110/696) of the differentially expressed genes. In total 26 KEGG terms were enriched in blastospores of which 16 are exclusively found among up-regulated genes in blastospores (Table 2, Additional file 4 and 5) and only a single KEGG-pathway was exclusively and significantly enriched among up-regulated in hyphae (ko00513: *Various types of Nglycan biosynthesis)* (Additional file 5).

**Table 2:** KEGG pathway terms significantly enriched among up-regulated genes in blastospores (Additional file 5)

KEGG	Pathway
ko00010	Glycolysis / Gluconeogenesis
ko00071	Fatty acid degradation
ko00190	Oxidative phosphorylation
ko00260	Glycine, serine and threonine metabolism
ko00310	Lysine degradation
ko00340	Histidine metabolism
ko00350	Tyrosine metabolism
ko00360	Phenylalanine metabolism
ko00380	Tryptophan metabolism
ko00410	betaAlanine metabolism
ko00620	Pyruvate metabolism
ko00630	Glyoxylate and dicarboxylate metabolism
ko01100	Metabolic pathways
ko01110	Biosynthesis of secondary metabolites
ko01130	Biosynthesis of antibiotics
ko01200	Carbon metabolism

To complement these gene set enrichment analyses, we further analyzed functional annotations of the differentially expressed gene set in relation to our five hypotheses outlined above, which we detail in the paragraphs below.

### Cellular growth and DNA homeostasis

We analyzed the putative function of genes differentially expressed between blastospores and hyphae for signatures of cellular growth and DNA homeostasis. In hyphae, we found six out of 13 significantly enriched GO terms in the biological processes category, which were related to cell division and DNA activities. These terms represent *DNA replication* (GO:0006260), *DNA translation* (GO:0006412), *DNA recombination* (GO:0006310), *DNA repair* (GO:0006281), *cell division* (GO:0051301) and *telomere maintenance* (GO:0000723) (Figure 5B). This is also evident in the significantly enriched KEGG-pathways in hyphae such as *DNA replication* (ko:03030), *DNA Mismatch repair* (ko:03430), *Base excision repair* (ko:03410), *Homologous recombination* (ko03440), *Nucleotide excision repair* (ko:03420) and *Pyrimidine metabolism* (ko:00240) (Additional file 4).

The increased DNA-related activity could indicate actively growing cells, and we therefore also identified for enriched GO terms in hyphae involved in fungal growth and cell proliferation, like *microtubule* (GO:0005874), *microtubule motor activity* (GO:0003777) and *microtubule-based movement* (GO:0007018). Microtubules represent polymers of tubulins that are components of the cell cytoskeleton that undergo rearrangements during cell growth. Some of the significantly enriched pfam terms found in hyphae is in concordance with these findings, such as the kinesin motor domain (PF00225) that function in proteins that moves along microtubules and is related to mitosis, meiosis and transportation of cellular components, and the protein kinase domain (PF00069), involved in cellular division, differentiation, and cytoskeletal rearrangement. Additionally, we found two genes, exclusively and highly expressed in hyphae involved in conidiophore formation and differentiation of conidiation structures, conidiospore surface protein (log_2_ fold change -6.3, FDR-*pvalue* < 0.001, KID70618) and conidiation-specific protein (log_2_ fold change -6.6, FDR-*pvalue* < 0.001, KID63680) (Additional file 3).

To maintain active growth, heterotrophic organisms like fungi need to break down nutritional substrates into smaller molecules that can be absorbed into the cell. In hyphae, we observed a high number of up-regulated genes involved in transportation of substances in membrane-bound vesicle (Figure 5B). These findings are illustrated by the significantly enriched GO term: *vesicle-mediate-transport* (GO:0016192) and pfam term: *Sugar (and other) transporter* (PF00083) in hyphae. In blastospores, we similarly found the significantly enriched GO term *Transmembrane transport* (GO:0055085), which indicates active transport of substance across external or intracellular membranes. The main metabolic pathway that provides energy for fungal cells is glycolysis and the TCA-cycle. These pathways were enriched in blastospores (FDR-*pvalue* < 0.25), emphasizing that genes facilitating an increased metabolic rate and energy consumption are up-regulated in blastospores compared to hyphae. Enrichment in blastospores of the glyoxylate and dicarboxylate metabolism pathways (KEGG: ko00630, FDR-*pvalue* < 0.25) further supports this inference (Additional file 4). This pathway is important for assimilation of alternative carbon sources like two-carbon substances and fatty acids. Specifically, two well-known glyoxylate cycle intermediates, isocitrate lyase (ICL, pfam: PF00463) and malate synthase (MLS, pfam: PF01274), that convert 2-carbon compounds like acetate and hydrolytic products of fatty acids (Padilla-Guerrero *et al.* 2011) were significantly up-regulated in blastospores (Two ICL genes in *M. anisoplaie* ARSEF 549, log_2_ fold change 5.4 and 1.8, FDR-*pvalue* < 0.001, KID66430 and KID66042, respectively, and MLS, KID70056, log_2_ fold change 3.0, FDR-*pvalue* < 0.001).

### Cell wall metabolism

**Figure 6:**
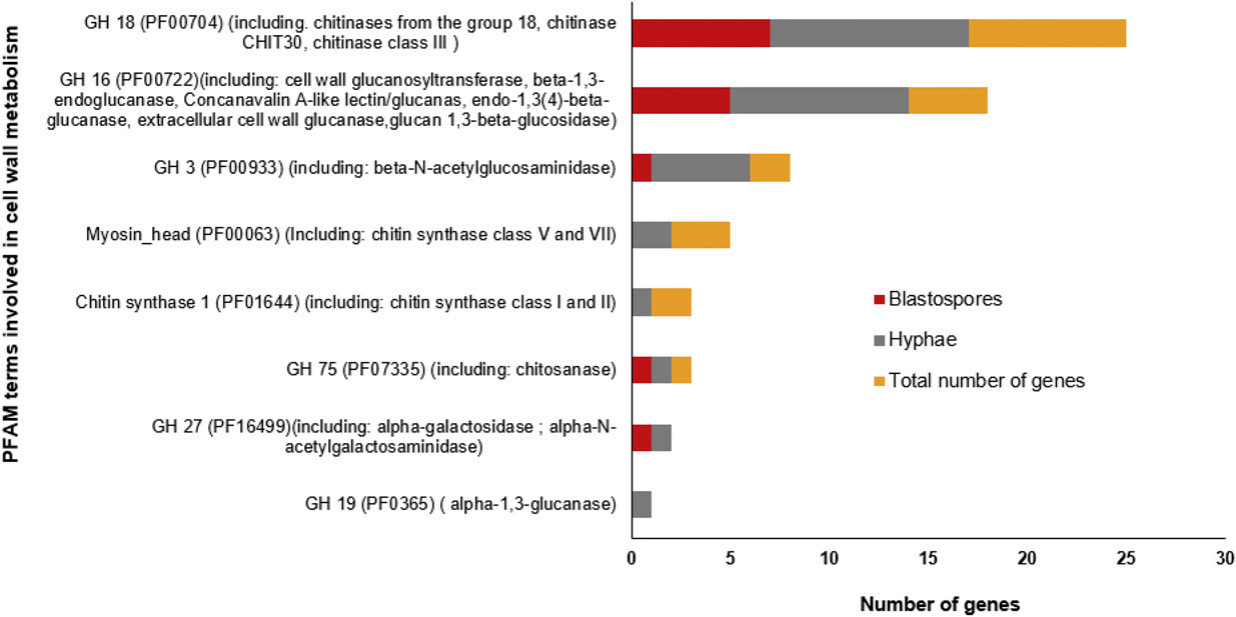
Number of genes with PFAM terms involved in cell wall metabolism. Up-regulated genes are represented by dark red bar for blastospores and gray bars for hyphae, FDR-*pvalue* < 0.001 (Additional file 6).

The main components of Ascomycete cell walls are polymers such as chitin, that make up the inner layer of the wall, and alpha/beta glucans and galactomannoproteins that comprise the gel-like polymers in the outer cell wall layer (Gow 1995). Differences in cell wall composition between *M. anisopliae* blastospores and hyphae was evident from the significant enrichment in hyphae of the GO category *carbohydrate metabolism* (GO:0005975) and KEGG pathway *Various types of Nglycan biosynthesis* (ko:00513) (Additional file 4). These classifications contain many genes related to cell wall metabolism, and we therefore expected to find gene expression differences in the glycoside hydrolases (GH), that synthetize or degrade cell wall components (Figure 6, Additional file 6). Enzymes in GH family 18 (PF00704), GH family 16 (PF00722), and GH family 3 (PF00933) were the largest and most differentially regulated group of GH’s between blastospores and hyphae (Figure 6). We identified 25 GH family 18 genes that includes chitinases from group 18 and class III, which degrade chitin and were expressed both in hyphae and blastospores (Figure 6). The second largest group of GH in *M. anisopliae* is GH family 16, which include enzymes involved mainly in degradation of cell wall polymers of glucans (Figure 6). In blastospores, glycoside hydrolases that include beta-1,3-endoglucanase (KID71664) and Concanavalin A-like lectin/glucanas (KID71072) were up-regulated, whereas in hyphae cell wall glucanosyltransferase, endo-1,3(4)-beta-glucanase, extracellular cell wall glucanase, glucan 1,3-beta-glucosidas and one gene assigned to Concanavalin A-like lectin/glucanase (KID68560) were up-regulated (Figure 6, Additional file 6). The essential component of fungal cell walls, chitin, is synthesized by chitin synthases and members of GH family 3 (PF00933), which were differentially regulated between blastospores and hyphae (Figure 6, Additional file 6). Chitin synthase is activated by N-acetylglucosamine and uses Uridin diphospho-N-acetylglucosamine (UDPGlcNAc) to produce chitin (Gow 1995, Elieh-Ali-Komi and Hamblin 2015). That chitin synthesis is primarily taking place in hyphae is further supported by the chitin synthase enzymes Chitin synthase 1 (PF01644), including chitin synthase class I and II, and Myosin_head (PF00063), including chitin synthase class V and VII, which were up-regulated in *M. anisopliae* hyphae but not in blastospores (Figure 6, Additional file 6). Analysis of the GH family 27 (PF16499) showed that alpha-galactosidase and alpha-N-acetylgalactosaminidase were up-regulated in blastospores and hyphae, respectively, whereas GH family 19 (PF0365) alpha-1,3-glucanases were exclusively up-regulated in hyphae (Figure 6, Additional file 6).

Specific cell wall proteins such as hydrophobins and adhesin are important for creating an hydrophobic surface that can provide protection to fungal cells and adherence to substrate or host (Bidochka *et al.* 1995, Silva *et al.* 2010), exemplified by the adhesin proteins MAD 1 (KID69933) and MAD 2 (KID69968) that were highly expressed in hyphae (log_2_ fold change 4.0 and 7.8, FDR-adjusted *P* < 0.001, respectively). Out of four genes encoding hydrophobin proteins in the *M. anisopliae*, one was significantly expressed in blastospores (KID65917) and another hydrophobin gene in hyphae (KID65291) (log_2_ fold change 4.1 and log_2_ fold change 6.1, FDR-adjusted *P* < 0.001, respectively)

### Cellular respiration and oxidative stress

Fungal respiration processes appear to be highly activated in blastopores produced under liquid growth conditions, as exemplified by the significantly enriched GO categories: *tricarboxylic acid cycle* (GO:0006099), *glycolytic process* (GO:0006096), and *ATP synthesis coupled proton transport* (GO:0015986) (Figure 5a). The pfam domains representing mitochondria protein family (PF00153) and Acyl-CoA dehydrogenase protein family domains (PF00441, PF02770, and PF02771) were similarly enriched among up-regulated genes in blastospores (Additional file 4). The mitochondrial respiratory mechanism constitutes the main intracellular source of reactive oxygen species (ROS). However, ROS accumulation within the cell trigger the cell responses to oxidative stress, which was evident in blastospores by the significant enrichment of GO category *response to oxidative stress* (GO:0006979) (Figure 5a).

### Expression of virulence factors

Because blastospores and hyphae differ in their insect infectivity, we investigated whether there were expression differences in pathogenicity-related genes between hyphae and blastospores. Certain protease enzymes are important for entomopathogenic fungi during penetration of the insect cuticle, and especially the subtilisin-like serine proteases (pfam: PF00082) are well known to be implicated in entomopathogenicity. Two out of 49 genes with PF00082 in the *M. anisopliae* genome were up-regulated in hyphae, the proteinase K-like, Pr1K (log_2_ fold change: -8.5, FDR-adjusted *P* < 0.001, KID64661) and subtilisin-like serine protease, Pr1C (log_2_ fold change: -4.6, FDR-adjusted *P* < 0.001, KID70176). In blastospores, only a single but different subtilisin-like serine protease Pr1C were up-regulated (log_2_ fold change: 4.2, FDR-adjusted *P* < 0.001, protein product: KID65298) (Additional file 8). Another group of proteases well known to be involved in pathogenicity of fungi are trypsin-like serine proteases (pfam:PF00089) (Dubovenko *et al.* 2010). Ten trypsin-proteases, out of 24 genes in the ARSEF 549 *M. anisopliae* genome containing domain PF00089 were expressed in hyphae, and four of them significantly and highly expressed (log_2_ fold change – 6.6, - 7.8, -5.2, -7.2, FDR-*pvalue* < 0.001, protein products: KID60022, KID63483, KID70241 and KID70499, respectively), whereas no trypsin-proteases were differentially expressed in blastospores (Additional file 8).

When entomopathogenic fungi grow inside insects they have to cope with the host immune response in the hemolymph. Fungi overcome host defense either by developing cryptic growth forms, like blastospores, that are partially masked from host defense responses or by producing immunomodulating molecules that suppress the host defense (Bidochka *et al.* 1995). Blastospores of *M. robertsii* express the collagen-like protein MCL1, which is a well know protein that provides an antiadhesive protective coat that masks beta-glucan components of the cell wall and hinders detection by the host hemocytes (Wang and St Leger, 2006). In our in-vitro experiments, the two identified MCL1 proteins in *M. anisopliae* (protein product: KID63518 and KID71631) were both up-regulated in blastospores, and one of them (KID63518) were highly expressed (log_2_ fold change 8.2, FDR-adjusted *P* < 0.001). Phosphoketolase, is an essential enzyme in the phosphoketolase pathway involved in sugar metabolism and required for full virulence in *Metarhizium* spp. This enzyme is usually highly expressed by *Metarhizium* species when grown in trehalose-rich insect haemolymph but poorly induced by insect cuticle (Duan *et al.* 2009). Here we found a phosphoketolase (protein product: KID62449) up-regulated in blastospores (log_2_ fold change 3.8, FDR-*pvalue* < 0.001).

### Secondary metabolites

**Figure 7:**
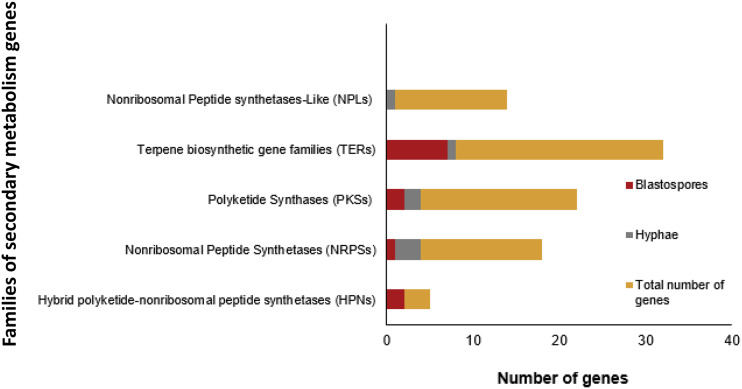
Number of genes in families of biosynthetic genes involved in secondary metabolism of *Metarhizium*. Up-regulated genes are represented by dark red bar for blastospores and gray bars for hyphae, FDR-*pvalue* < 0.001, log_2_FC > 4 (Additional file 9).

Many fungi produce high amounts of secondary metabolites important for pathogenicity, and one of the best known types of secondary compounds produced by *M. anisopliae* during fermentation are destruxins. In the present experiments both blastospores and hyphae expressed the gene destruxin synthetase (KID59658, gene: MAN_10464; Mean (± SE) of sample normalized count data for hyphae: 1522 ± 20 and blastospores: 1476 ± 67) (Additional file 1), but there was no difference in expression between the two fungal structures (log_2_ fold change -0.04, FDR-adjusted *P* = 0.61). The KEGG pathway *Biosynthesis of secondary metabolites* (ko01110) were enriched in blastospores and a higher number of significantly expressed secondary metabolite related genes were observed in blastospores (n = 12) compared to hyphae (n = 7) indicating that blastospores at time of harvest from the liquid medium produce more secondary metabolites than hyphae (Figure 7). A gene annotated as Non-ribosomal Peptide synthetase-like (NPLs) (*MAN_01071*/ KID71472) where exclusively expressed in hyphae, whereas two genes related to Hybrid polyketide-nonribosomal peptide synthetases (HPNs) (*MAN_01651*/KID69137, *MAN_09390*/ KID61106) where exclusively expressed in blastospores (Figure 7). Although expression occur in both blastospores and hyphae, Nonribosomal Peptide Synthetases (NRPSs) where primarily expressed in hyphae (n = 3), whereas Terpene biosynthetic gene families (TERs) where primarily expressed in blastospores (N = 8) (Figure 7, Additional file 9).

Cytochrome P450 is a large group of proteins that among other functions are involved in conversion of hydrophobic intermediates of primary and secondary metabolic pathways and detoxification processes that sustain fungal growth under stressing conditions. Out of 103 Cytochrome P450 genes (PF00067) in M. anisopliae, we found 34 genes differentially expressed between blastospores and hyphae (log_2_ fold change >4 or < -4, FDR-adjusted *P* < 0.001) (Additional file 11). These where divided evenly with 16 up-regulated in blastospores and 17 up-regulated in hyphae. Many of these Cythocrome P450 genes were among the most highly expressed genes as exemplified by their presence on the list of the top 50 up-regulated genes in blastospores (Additional file 7)

## Discussion

Here we compared the transcriptomic profile of blastospores and hyphae induced by growth in liquid and solid medium, respectively, of the dimorphic entomopathogenic fungus *M. anisopliae*. Hyphae and blastospores were grown under identical nutritional conditions and the media thus only differed in oxygen levels, water composition, viscosity and agitation. In total, 6.4% (n = 696) out of the 10,981 predicted genes in *M. anisopliae* were significantly differentially expressed between blastospores and hyphae with a fold-change > 4. Functional annotation and gene enrichment analyses highlighted that the main physiological processes that were up-regulated in the single-celled yeast-like blastospores during liquid fermentation were: oxidative stress, amino acid metabolism, respiration processes, transmembrane transport and production of secondary metabolites. In contrast, hyphae had molecular signatures of increased growth metabolism and cell wall re-organization, which underlines the different growth morphology of *M. anisopliae* blastospores and hyphae.

### Differential growth of hyphae and blastospores

Hyphae and blastospores show clear differences in their pattern of growth. Hyphae exhibit polarized growth whereas blastospores grow isotropically until cell division by budding (Fang *et al.* 2010). The three principal components of polar growth characteristic for fungal hyphae, are vesicles, responsible for the enzyme supply for cell wall synthesis, the cytoskeleton that provides a scaffold for vesicle transport and cell wall polymers that sets the shape of the cells (Gow 1995). Several genes from all three components were highly expressed in *M. anisopliae* hyphae (Figure 4 and 5). This is similar to other pathogenic dimorphic fungi (Table 3), and consistent with hyphal cell walls being more rigid than single-celled yeast-like blastospores in *Metarhizium*.

**Table 3:** Comparison of major biological and physiological processes of metamorphosis in key pathogenic dimorphic fungi

	Fungus	Disease	Pathogenicity and virulence	Morphogenetic induction	Cell-wall organization	Oxidative stress	Secondary metabolism	Reference
***Insect pathogens***	***Metarhizium anisopliae (Ascomycota*; *Sordariomycetes*; *Hypocreales*; *Clavicipitaceae)***	Green muscardine	Infective propagules are conidia and single-celled yeast-like phase (blastospores). Blastospores have been shown to be more virulent that aerial conidia toward arthropodes.	Blastospores are induced during growth inside insects and in liquid media with agitation. Unknown inducing conditions (Suggested factors: high osmotic pressure and oxidative stress)	Numerous enzymes involved in cell-wall synthesis and breakdown are differentially regulated between dimorphic forms. Blastospores has thinner cell walls then hyphae and it is composed by glucans and chitin.	Blastospores experience oxidative stress *in vitro* and produces enzymes such as catalases and peroxidases.	Nonribosomal peptides, Polyketides and Terpenoids are differnetially expressed between growth forms. The most well known are Destruxins and Cytochalasins.	[This study, Wang and Leger 2006, Sbaraini *et al.* 2016 ]
	***Beauveria bassiana (Ascomycota*; *Sordariomycetes*; *Hypocreales*; *Cordycipitaceae)***	White muscardine				Blastospores experience oxidative stress *in vivo* and produces catalases, intracellular polyol accumulation, activation of high osmolarity glycerol (Hog1) MAP‐kinase pathways and superoxide dismutases (SODs).	Polyketides like oosporein, bassianin and tenellin, nonribosomally peptides like beauvericin, bassianolides and beauveriolides are differentially expressed between growth forms.	[Bidochka *et al.* 1987, Tartar *et al.* 2005, Xiao *et al.* 2012, Wang *et al.* 2013, Ortiz-Urquiza and Keyhani (2016)]
***Plant patogens***	***Ophiostoma novo-ulmi*** *(Ascomycota*; *Sordariomycetes*; *Ophiostomatales*; *Ophiostomataceae)*	Dutch elm disease.	Spread by elm beetles from family Curculionidae. The fungus invades vascular system of trees. Both hyphae and yeast play a role in pathogenicity.	Yeast-phase is induced during growth inside of the vascular system of trees. Nitrogen source, proline aminoacid, salicylic acid (cyclooxygenase inhibitor), and oxylipins induce yeast-phase *in vitro*.	Chitin synthases and aminoglycan metabolite process are highly expressed in mycelium. Glycoside hydroxylases and glycosyltransferases are expressed differently in yeast and mycelium.	Yeast phase show an increase in catalase production and increase in oxidation-reducing processes.	Produces nonribosomal peptides, some polyketides produced only in mycelial phase, siderophore biosynthesis, fujikurin-like compounds produced in both yeast and mycelial phases.	Jensen *et al.* 1992, Naruzawa and Bernier, 2014,Nigg *et al.* 2015, Sbaraini *et al.* 2017
	***Ustilago maydis*** *(Basidiomycota*; *Ustilaginomycetes*; *Ustilaginales*; *Ustilaginaceae)*	Corn smut	Yeast phase is saprophytic while hyphae are pathogenic induce formation of host tumors in maize.	Mating interaction, nutrient starvation, Ph, pheromones	β-1,6-glucan synthesis, N-glycosylation membrane proteins, hydrophobins and chitin synthase, glycosidases and others polymers are differentially expressed between filamentous and yeast phases.	Hyphae experience oxidative stress during proliferation in host tissue and respond to host ROS by producing ROS‐detoxifying enzymes phospholipase and Superoxide dismutase.	Many polyketides and non-ribosomal peptides, siderophores, indole pigments, ferrichrome, Pityriacitrin, and ustilagic acid are differentially expressed between growth phases.	Banuett and Herskowitz, 1994, Sánchez-Martínez and Pérez-Martín, 2001, Bölker *et al.* 2008, García-Pedrajas *et al.* 2010, Robledo-Briones and Ruiz-Herrera, 2013, Kunst *et al.* 2016

***Human pathogens***	***Candida albicans*** *(Ascomycota*; *Saccharomycetes*; *Saccharomycetales*; *Saccharomycetaceae*	Candidiasis	Commensal fungus. Both yeast and filamentous phase are important for full virulence	Temperature, Ph, nutrient deprivation, quorum sensing	Differences in composition of cell walls between yeast and mycelial phase. High amount of chitin in hyphae, while the amount of glucans and mannoproteins are similar between both fungal structures.	Fungus experiences oxidative stress induced by macrophages during proliferation in host tissue. Produces catalases, gluthatione peroxidases and other antioxidants in response.	Produce many secondary metabolites like farnesol that inhibits transition from the yeast to hyphae. Farnesol protect Candida from oxidative stress.	Sánchez-Martínez and Pérez-Martín, 2001, Albuquerque and Casadevall (2012), François *et al.* 2013, Ruiz-Herrera *et al.* 2006, Whiteway and Bachewich, 2007, Dufour and Rao, 2011, Dantas *et al.* 2015
	***Paracoccidioides brasiliensis*** *(Ascomycota*; *Eurotiomycetes*; *Onygenales*; *Ajellomycetaceae)*	Both yeast and filamentous phase are important for virulence. Hyphae undergo thermal-induced differentiation into a yeast phase inside host lungs.	Temperature	Reorganization of membrane lipids and carbohydrate polymers. Increase in chitin content in yeast. α-glucan and β-glucan as main polysaccharides in the cell wall of yeast and hyphae, respectively. Hydrophobins are mycelium specific.	Oxidative stress in yeast phase results in production of superoxide dismutases, catalase, and glutathione peroxidase thiol-specific antioxidant gene (TSA1) protects against ROS and RNIs.	Genes encoding enzymes involved in terpenoid and melanin biosynthesis are present.	Albuquerque *et al.* 2004, Nunes *et al.* 2005, Tomazett *et al.* 2005,

	***Talaromyces marneffei*** *(Ascomycota*; *Eurotiomycetes*; *Eurotiales*; *Trichocomaceae)*	Penicilliosis	Filamentous phase is saprophytic while yeast-like cells are pathogenic. Causes disease especially in immunocompromised patients. Melanins in yeast form protects from host immune system.	Temperature	Several enzymes involved with changes in cytoskeletal organization during morphogenesis. Cell wall composition of yeast and mycelia are different.	Fungus experience oxidative stress induced by macrophages during proliferation in host tissue. It produces superoxide dismutase and catalases during macrophage infection and yeast growth.	Secondary metabolism in both growth phases. Polyketides involved in biosynthesis of pigments like melanin during yeast growth and red pigments in mycelium.	Cooper and Vanittanakom, 2008, Boyce *et al*. 2013, Tam *et al.* 2015


The main components of Ascomycete cell walls are chitin, alpha/beta glucans and galactomannoproteins (Gow 1995). These polymers are synthesized and broken down by a broad group of enzymes named Glycoside hydrolases (GH). One group of GHs important for cell wall modulation and involved in chitin degradation, are the chitinases that break β-1,4-linkages. These enzymes are found in two GH families GH18 and GH19 with distinct catalytic mechanisms (Junges *et al.* 2014, Mondal *et al.* 2016). The GH18 family chitinases are the most common in fungi (Hartl *et al.* 2012), and we discovered three GH18 chitinases significantly up-regulated in hyphae while only a single GH18 chitinase was significantly up-regulated in blastospores (Additional file 6). This is consistent with previously reported low activity of chitinases in *M. anisopliae* blastospores compared to mycelium (Junges *et al.* 2014). However, in blastospores a beta-1,3-endoglucanase (GH16), which is involved in degradation of glucan polymers was highly expressed (Additional file 6). The differential expression of cell-wall related GH enzymes (Figure 6), is consistent with the *M. anisopliae* blastospore cell walls being thinner than cell walls in aerial and submerged conidia (Leland *et al.* 2005). This is likely explained by the submerged growth of blastospores in liquid medium allowing increased flexibility and permeability of the cell wall (Wang *et al.* 2005). These cell wall modifications enhance nutrient flux and our results suggests they are induced in response to different nutritional and environmental conditions.

Seven distinct classes of chitin synthases belonging to two families have been identified in fungi based on amino acid sequences (Morozov and Likhoshway 2016). Here we found classes I, II, V and VII expressed in hyphae and blastospores. The class V and VII are primarily involved in synthesis of chitin at the tip of growing hyphae and during conidiogenesis and not present in most yeast species (Fernandes *et al.* 2016). When infectious conidia are produced by *M. anisopliae* they develop from conidiophores formed in the hyphal growth phase and not directly from blastospores. Consistent with this, we observed up-regulated expression of the enzyme beta-N-acetylglucosaminidase (gene *MAN_04359*, KID67601) involved in chitin synthesis pathways and genes related to conidiophore and conidiogenesis differentiation exclusively in hyphae.

The synthesis of new biological material in hyphae correlates with increased activity of cell wall metabolism, but also with high activity of genes associated with DNA and carbohydrate metabolism. However, these findings do not agree with the fact that the main energetic metabolic pathways like: TCA-cycle, glycolysis and glyoxylate cycle are all up-regulated in blastospores, as well as amino acid metabolic pathways. The glyoxylate cycle is activated by many fungi when non-fermentable carbon sources and 2-carbon compounds are provided (Lorenz and Fink 2001). *M. anisopli*ae species up-regulate the glyoxylate cycle during growth within insect hemocytes, but represses the glyoxylate cycle when the fungus evades hemocytes and proliferate in the hemolymph (Padilla-Guerrero *et al.* 2011). This suggests that *M. anisopliae* can hydrolyze and extract energy from intracellular lipids during growth within insect hemocytes (Padilla-Guerrero *et al.* 2011). In our experiment, *M. anisopliae* blastospores were grown in rich glucose medium (140g/L) with two protein sources, yeast extract and cornsteep liquor. The finding that the glyoxylate cycle, glycolysis and TCA-cycle is up-regulated in blastospores, indicates that energy for cell metabolism in this stage comes from different sources of substrate and not only glucose. The glyoxylate cycle is known to be activated in bacteria under oxidative stress (Ahn *et al.* 2016), and although we found that blastospores are under oxidative stress, the correlation with the glyoxylate cycle is to the best of our knowledge unknown in fungi.

When hyphae are growing and multiplying (*i.e.*, consuming energy and building new biological material), we expected the major metabolic cycles to be up-regulated in hyphae and not in blastospores. Instead, the observed transcriptome pattern indicates that not all the energy produced by blastospore metabolic activity is converted into new biological material in the form of new growth, otherwise we would have expected DNA associated activities to be equally up-regulated in blastospores and hyphae. These results could be partially explained by continuous polarized growth of hyphae as long as conditions are favorable, whereas yeast-like growth of blastospores is determined and ends when development is complete (Gow 1995). Microscopic analysis of slides from each biological replicate at the moment of RNA extraction revealed that few blastospores were actively replicating. This could be due to beginning physio-chemical changes in the media or that some blastospores had reached the final size and stopped cell division when these cells were harvested from the medium at the end of the exponential phase. Another perspective is that the formation of hyphae is induced by the fungus so that the chances of finding nutrients increase. Therefore, the intense metabolism of hyphae, involving secretion of hydrolytic enzymes, may reflect a constitutive response of hyphae in seek for nutrients in their natural habitat. These differences in growth correlates with low chitinase expression in blastospores compared to hyphae, where in contrast hyphal growth involves constant degradation and synthesis of the external and internal septal cell walls. Increased expression of cell wall organization proteins in hyphae has also been reported for other dimorphic fungal pathogens such as the human pathogen *Paracoccidioides brasiliensis* (Andrade *et al.* 2006) and the phytopathogenic fungus *Ustilago maydis* (Robledo-Briones and Ruiz-Herrera 2013) when compared to their yeast phases, respectively. The composition of cell walls is fundamentally different between yeast and mycelial forms in many fungi (Domer *et al.* 1967, Kanetsuna and Carbonell 1970, Chattaway *et al.* 1973 and table 3), and our RNAseq data are consistent with this also being the case between *M. anisopliae* blastospores and hyphae.

### Oxidative stress in blastospores during submerged growth

Oxidative stress is caused by intracellular accumulation of reactive oxygen species (ROS) or changes in cellular redox stability. External environmental causes for ROS formation are: ionizing radiation, visible light, temperature shifts, oxygen exposure and UV radiation. Cellular defense against oxidative stress involves enzymatic and non-enzymatic detoxification mechanism aiming to remove ROS and keep the redox balance (Mager *et al.* 2000). In our study, the high agitation speed (350rpm) provided to culture flasks and high glucose concentration of the medium (140g/L) result in high dissolved oxygen levels (Giese *et al.* 2014, Mascarin *et al.* 2015), which likely expose blastospores to increased oxygen levels that trigger the formation of ROS. Another possible explanation for this physiological response would be the constitutive expression of antioxidant enzymes as a defense mechanism against the stressful conditions in the hemolymph of insects where blastospores are naturally produced (Dubovskii *et al.* 2010a, 2010b). A ROS-mediated immune response toward the host is supported by increased expression of antioxidant catalases enzymes involved in the response to oxidative stress (GO:0006979) in blastospores. These catalase enzymes break down the ROS hydrogen peroxide (H_2_O_2_) into H_2_O and O_2_, consistent with blastospores being more stressed by ROS than hyphae in our experiments.

The mitochondrial respiratory chain constitutes the main intracellular source of ROS in most tissues (Turrens 2003). Indeed, the high activity of the TCA-cycle and respiration processes in blastospores could also be related to ROS production. An increase in intracellular ROS levels have been shown to induce morphogenesis of other fungi (Table 3), and also in propagules under aerated liquid culture conditions such as in the formation of microsclerotia in the entomopathogenic fungus *Metarhizium rileyi* (Song *et al.* 2013), hyphal to yeast transitions in *P. brasiliensis* (Nunes *et al.* 2005), and the formation of sclerotia in phytopatogenic fungi (Papapostolou and Georgiou 2010). These findings could indicate a role for ROS in blastopore morphogenesis, but the high levels of intracellular ROS would need to be tightly regulated because of the toxicity of radicals. Proteins from the versatile group of Cytochrome P450 are among other functions also involved in detoxification of ROS, and for example, we observed comparatively more Cytochrome P450’s and monooxygenases among the top 50 most up-regulated genes in blastospores. This list also contained the important copper oxidase enzyme laccase, which can provide protection against oxidative stress caused by oxygen radicals in fungi (Thurston 2019). The laccase of the white rot fungus, *Pichia pastoris*, significantly enhances scavenging of intracellular H_2_O_2_ and lipid oxidative damage by stimulating production of glutathione-based antioxidants (Yang *et al.* 2012). Glutathione is an important antioxidant in fungi, and we found a glutathione S-transferases among the top 50 most up-regulated genes in blastospores (log2FC: 10.5, FDR-*pvalue* < 0.001, protein product: KID61097). This enzyme is involved in detoxification of reactive electrophilic compounds by catalyzing their conjugation to glutathione, indicating that laccase has an important indirect role for protecting blastospores against oxidative stress. The transcriptome profile of blastospores indicate that ROS may be involved in blastospore formation but also as a stressor that force blastospores to constantly try to maintain cell homeostasis and probably excrete cytotoxic components produced during oxidative process. The response to oxidative stress may be a constitutive condition posed by the liquid culture medium that to some extent mimic an insect hemolymph environment.

The transcriptome profiles highlighted differences in the transport of substances between hyphae and blastospores, where transmembrane transport was more active in blastospores whereas vesicle transport of substance was more pronounced in hyphae. Increased transport activity across the outer membrane has also been observed in the yeast phase of three human fungal pathogens: *Histoplasma capsulatum* [3], *Paracoccidioides brasiliensis* (Bastos *et al.* 2007), and *Penicillium marneffei* (Yang *et al.* 2014). The outer membrane transporters functions in the absorption of nutrients, export of toxic molecules, maintenance of cell turgor, cell development, and maintenance of ion and pH homeostasis (Kschischo *et al.* 2016). A high number of functional categories related to amino acid metabolism and respiration metabolism were significantly up-regulated in blastospores suggesting that the high activity of membrane transporters in blastospores could be partially explained by increased amino acid metabolism, glycolytic processes and the export of toxic molecules in response to oxidative stress.

Although transmembrane transport is present in hyphae, genes involved in vesicle-mediated transport were significantly up-regulated in hyphae compared to blastospores. During mycelial growth, not all hyphal cells in the mycelium are in direct contact with the substrate and resources are usually reached at the tips of hyphae, sometimes following branching of the hyphae depending on fungal species. Different from the multi-cellular hyphae, yeast-like growth is characterized by the cells being in constant direct contact with the substrate and resources are reached by growth (Gow 1995). The vesicle mediated transport play an important role for delivering nutrients to cells that are not in direct contact with the substrate, in order to supply enzymes for cell wall synthesis and to transport material across cell wall (Casadevall *et al.* 2009). Thus, the vesicle-mediated transport is potentially more important for mycelium development then for the yeast-like phase of blastospores in *M. anisopliae*.

### Secreted metabolites and virulence related enzymes

The main group of genes involved in secondary metabolite production up-regulated in blastospores were the terpenoids (Figure 7). Four (M-TER10, M-TER11, M-TER26 and M-TER31) out of seven terpenoid families found up-regulated in blastospores (Figure 7), belong to a conserved cluster that closely resemble those involved in indole diterpene production in other Ascomycetes (Wang *et al.* 2016). Some indole diterpenes are known to have insecticidal effects through ion channel modulation (Knaus *et al.* 1994 and Smith *et al.* 2000), and indole-diterpenes from sclerotia of species in the genera *Aspergillus* and *Penicillium* have biological activity against insects (Lo *et al.* 2012). Although indole diterpenes have been isolated from Clavicipitaceaen fungi, their chemical details have not been worked out in *Metarhizium* (Donzelli and Krasnoff 2016). Two other families of terpenoides expressed in blastospores, M-TER33 (terpene cyclase) and MTER43 (UbiA prenyltransferase) are homologs to *Aspergillus nidulans* genes ausL and ausN, which are responsible for catalyzing terpene cyclization and C-alkylation of the 3,5-dimethylorsellinic acid in the meroterpenol austinol pathway (Lo *et al.* 2012 and Young *et al.* 2015). Only a single tryptophan dimethylallyltransferase terpene (M-TER29) were up-regulated in hyphae (Figure 7), which catalyses the first step of ergot alkaloid biosynthesis that are mycotoxins toxic to animals and are important in pharmaceutical industry (Young *et al.* 2015). A similar pattern of extensive expression in blastospores compared to hyphae was also seen for hybrid polyketide-nonribosomal peptide synthetases (PKS-NRPS) (Figure 7). The PKS-NRPS, M-HPN2, expressed in blastospores correspond to NGS1 synthetase related to biosynthesis of the NG-391 in *M. robertsii* (Donzelli *et al.* 2010). NG-391 is a mutagenic mycotoxin first identified in *Fusarium* species. This toxin is highly produced in the exponential growth phase of *M. robertsii* mycelium, but detected in low quantities during the stationary growth phase in liquid culture and in early phases of insect infection (Donzelli *et al.* 2010). NG-391 is not detected in *M. robertsii* conidia and is suggested to be developmentally regulated (Donzelli *et al.* 2010). Our finding is to the best of our knowledge the first to relate NGS1 expression in *M. anisopliae* blastospores when grown in liquid broth. Although an NGS1 knock-out mutant of *M. robertsii* did not reduce virulence of *M. robertsii* conidia toward *Spodoptera exigua* (Donzelli *et al.* 2010), the specific expression in blastospores is consistent with fungal growth related expression. Transcriptome patterns also indicated that blastospores may be producing terpenoid substances, because two polyketide synthases (PKSs) conserved within *Metarhizium* (M-PKS24 and M-PKS28) were up-regulated in blastospores (Figure 7), (Donzelli and Krasnoff 2016). These two PKSs have similar chemical functions with two polyprenyl transferases (M-TER43 and M-TER33), which we also found to be up-regulated in blastospores (M-TER43: log_2_ fold change 5.4, FDR-*pvalue* < 0.001 and M-TER3: log_2_ fold change 4.2, FDR-*pvalue* < 0.001). Several different biosynthesis gene families of secondary metabolites are up-regulated in blastospores, which is consistent with blastospores produced during liquid in-vitro growth expressing a distinct repertoire of secondary metabolites. This secondary metabolite transcriptome profile almost certainly plays a role in the high insect-pathogenic potential of blastospores.

Species of *Metarhizium* not only rely on secondary metabolites but also produces several secreted proteins that are important during pathogenesis. Especially, subtilisin-like serine proteases are important during conidial germination and appressorial penetration of the insect cuticle (St. Leger *et al.* 1996). In the present study, hyphae expressed two subtilisin-like serine proteases (Pr1C and Pr1K) and three trypsin-like serine proteases, whereas blastospores only up-regulated a single subtilisin-like serine protease (Pr1C). Since our experiments are exclusively performed during *in-vitro* growth, we interpret the expression of these proteases in blastospores but especially in hyphae, to be related with acquisition of proteinaceous nutrients from the substrate (Sowjanya Sree *et al.* 2008) and not related to virulence.

Our study revealed that blastospores produce the collagen-like protein MCL1 when grown in liquid culture medium (Additional file 1). The MCL1 protein provides an antiadhesive protective coat that masks beta-glucan components of the blastospore cell wall and thereby hinder hemocytes from killing the fungal cells during growth in insect hemolymph (Wang and St Leger 2006). Since this protein is also expressed in blastospores grown during *in-vitro* cultivation in liquid media with agitation, it supports that blastospores of *M. anisopliae* produced during *in-vivo* and *in-vitro* growth are comparable and that MCL1 does not require insect hemolymph to be induced.

Furthermore, the insect-pathogenic fungus *M. anisopliae* produces a group of mycotoxins named destruxins, which are crucial for colonization of the insect body and have insecticidal effects against many pests (Sowjanya Sree *et al.* 2008 and Hu *et al.* 2009). We found no difference in gene expression of the destruxins, both hyphae and blastospore express them in solid and liquid medium, respectively (KID59658, gene: *MAN_10464*, Additional file 1). These findings suggest that destruxins are partially constitutively expressed or have additional functions to pathogenicity. The production of destruxins in liquid culture is well known (Amiri-Besheli *et al.* 2000), but our data suggests that blastospores could be a potential candidate for exploring production of destruxins for toxicity studies. This might be especially relevant for the chemical and pest management industry, although the amount and the types of destruxins produced by blastospores and the chemical and biological characterization would need to be worked out first.

### Considerations for in-vitro production of M. anisopliae blastospores

The main fungal propagules used in biological control programs worldwide are conidia. However, commercial interest in industrial-scale blastospore production has increased lately due to numerous advantages of blastospores and the production process compared to industrial scale production of conidia on high-quality solid substrates such as rice. Our transcriptome study contributes to clarify some aspects of blastopore metabolism when grown in liquid culture, such as oxidative stress, can be one of the morphogenetic factors inducing the formation and maintenance of these cells in liquid medium. Therefore, it will be essential that blastospores produced in industrial bioreactors are provided with appropriate oxygen levels to maintain the desired metabolism profile. The oxygen level could be manipulated mainly by injection of oxygen or airflow inside of bioreactors or by increasing the rotation speed of liquid cultures. We have confirmed that the transcriptomic profiles corroborate a thinner cell wall of blastospores with less components compared to hyphal cell walls. These traits must be taken into account during blastospore manipulation for industrial processes, for example via formulations and drying process that decrease cell wall damage. Finally, we have shown that blastospores express genes for secondary metabolite synthesis, but very little is currently known and future studies to explore their role as insecticidal compounds could for example start with the group of indole terpenoids. In conclusion, we consider that blastospores can be industrially prospected, not only to make products for use in biological control programs but also for the industrial production of substances for other commercial use.
